# Supplementary material for: Diagnosing peri-implant disease using the tongue as a 24/7 detector
Source: Nat Commun. 2017 Aug 15;8:264. doi: 10.1038/s41467-017-00340-x (PMC5557808; doi:10.1038/s41467-017-00340-x)
Supplement: Supplementary file 1 — Supplementary Information [file 41467_2017_340_MOESM1_ESM.pdf]

Description of Supplementary Files

Title: Supplementary Information

Description: Supplementary Methods, Supplementary Figures, Supplementary Tables, and Supplementary References

Title: Peer Review File

Supplementary Methods

Supplementary MaterialsFmoc-Gln(Trt)-Wang resin and human neutrophil matrix metalloproteinases (MMPs) were from EMD Millipore Corporation (Billerica, MA). 5,6-Carboxyfluorescein (Cf) and all amino acid analogues used for Fmoc acid coupling strategy (solid phase peptide synthesis - SPPS) were purchased from VWR (Ismaning, Germany) unless noted otherwise. Fmoc-Rink-Amid-PEG-AM, Fmoc-Rink-Amid-AM and Fmoc-Rink-Amid-PS-AM resin were obtained from Iris Bio-Tech (Marktredwitz, Germany). 2-Chlorotrityl chloride resin (CTC) was from Chem-Impex Wood (Dale, IL). Fmoc-L-beta-azidoalanine (Fmoc-L-Dap(N3)-OH) was purchased from Iris BioTech or ChemPep (Wellington, FL). Poly(methylmethacrylate) (PMMA)-beads were obtained from PolyAn (Berlin, Germany).

Copper(I) sulphate (Cu_2SO_4), tris(2-carboxyethyl)phosphine (TCEP), tris[(1-benzyl-1H-1,2,3-triazol-4-yl)methyl]amin (TBTA), tertiary butanol, ethylenediaminetetraacetic acid (EDTA), sodium laurylsulphate (SDS), 4-aminophenylmercuric acetate (APMA), sodium hydroxide (NaOH), sodium chloride (NaCl), Trizma® hydrochloride (Tris-HCl), calcium chloride ($CaCl_2$), zinc chloride ($ZnCl_2$), BRIJ® 35, diethyl ether, dicyclohexyl carbodiimide (DCC), 4-dimethylamino pyridine (DMAP), 2-(diethylamino)-N-(2,6-dimethylphenyl)acetamide (lidocaine), 1-hydroxybenzotriazole hydrate (HOBt), piperidine, 4-(bromomethyl)benzoic acid, N,N-diisopropylethylamine (DIPEA), 1,2-ethanedithiol, leucine aminopeptidase (AP), microsomal from porcine kidney, L-eucin-p-nitroanilide, triisopropylsilane (TIS), acetic anhydrite, N-(2-aminoethyl)acetamid, 1-ethyl-3-(3-dimethylaminopropyl)-carbodiimide (EDC), N-hydroxysulfo-succinimide (Sulfo-NHS), Fmoc-3-azido-L-alanine (Fmoc-β-azido-Ala-OH), Denatonium benzoate (denatonium) (all from Sigma-Aldrich Chemie, Schnelldorf, Germany), tetrahydrofuran (THF), diethyl ether, dimethyl formamide (DMF), methanol, chloroform, hydrochloric acid, silica gel (all from Fisher Scientific, Schwerte, Germany), methyl 3-(bromomethyl)benzoate, methyl 4-(bromomethyl)benzoate (ABCR, Karlsruhe, Germany), ethanol (Carl Roth, Karlsruhe, Germany), α-cyano-4-hydroxycinnamic acid (Bruker Daltonics, Billerica, MA) diisopropyl carbodiimide (DIC), thioanisole, m-cresol and glycine ethyl ester hydrochloride (from Fluka, Buchs, Switzerland) were used without further purification. Aerosil was from Degussa (Düsseldorf, Germany) and magnesium stearate was from Euro OTC Pharma (Bönen, Germany). Tartaric acid was from AppliChem (Darmstadt, Germany).

Dichloromethane (CH_2Cl_2, DCM), acetonitrile (HPLC grade) and trifluoroacetic acid (TFA (HPLC grade)) were from VWR. The water was derived from an in-house demineralization system from Merck Millipore. All other chemicals used were at least of pharmaceutical grade and were purchased from Sigma-Aldrich (unless noted otherwise).

Synthesis of denatonium derivatives

Denatonium chloride was first synthesized by reaction of lidocaine with benzyl chloride [1]. To date, it is the most bitter substance known and the bitter taste can be detected at concentrations of 10 ppb [2]. Because of its lower toxicity as compared to the bitter molecules brucine [3] or strychnine it is widely used as an additive for denaturing alcohol, detergents, disinfectants or pesticides [4, 5].

Usually, the quaternization reaction of amino compounds such as lidocaine with benzyl halides will be performed in solvents like water [6]. Reactive benzyl halides for instance benzyl bromide even react at room temperature without any solvent [7]. Our research required the carboxylation of denatonium bromide for further possible coupling reactions. First experiments to synthesize carboxylated denatonium bromides in solution failed or resulted in small yields of desired product (**Supplementary Figure 1**). Precipitation of an undesired ammonium salt appeared at the reaction of lidocaine with 4-(bromomethyl)benzoic acid in THF. For prevention of undesired reaction products methyl ester was used. The further coupling reaction with lidocaine under microwave conditions using CEM Discover SP (CEM, Germany) in water yielded only 10% of product (**Supplementary Figure 1**).

During several experiments we found, that differently ring-substituted (halomethyl) phenyl derivatives react with lidocaine without solvent at room temperature (i.e. in dry state). Starting off these surprising findings, we included methyl 4-(bromomethyl)benzoate and methyl 3-(bromomethyl)benzoate in our studies (**Supplementary Figure 2**).The reactions are observable as a melting reaction. After 24 hours the product can be isolated as white powder by precipitation in THF whereas the para-substituted compound (**1**) was observed in 50 % and the meta-substituted compound (**2**) in 80 % yield. Hydrolysis of the methyl ester was performed in boiling ethanol with excess sodium hydroxide. Hydrochloric acid was added for neutralization.

The formed Na halide by-products had similar solubility properties as the carboxylated denatonium derivative and were both soluble in water and methanol. Therefore, the separation of the product was difficult and we changed the system to barium hydroxide and sulfuric acid. The precipitate of $BaSO_4$ was filtered off and after removing the solvent and treating with THF the product 3 was obtained in 90 % yield (**Supplementary Figure 3**)

The described reaction of lidocaine and bromomethylbenzoate was extended to other substrates (**Supplementary Figure 4**). Using methyl 2-(4-(bromomethyl)phenyl)acetate as starting material, the reaction occurred at room temperature. The product (**4**) was obtained at 55 % yield. The synthesis of compound (**5**) required two steps. The coupling of glycine ethyl ester hydrochloride to 4-(bromomethyl)benzoic acid was performed via carbodiimide chemistry. The resulting ethyl 2-(4-(bromomethyl)benzamido)acetate was coupled to lidocaine without solvent (i.e. by mixing in dry state). In this case, a temperature of 80 °C was necessary to start the reaction. The heating was lasted for 10 minutes. The raw product was precipitated with THF, resulting in a white product of 45 % yield (**Supplementary Figure 4**).

In conclusion we found a simple method for synthesizing modified denatonium derivatives for further possible coupling reactions. Quaternization reactions of lidocaine did not require solvents and proceeded at room temperature for most of the substrates.

Synthesis of compound 1

Lidocaine (1 g, 4.3 mmol) and methyl 4-(bromomethyl)benzoate (0.98 g, 4.3 mmol) were mixed at room temperature and reacted. After 24 hours, THF (10 mL) was added and the mixture was stirred until the raw product converted into a white powder. The product was filtered off, washed two times with THF and dried at 40 °C to afford 2-(2,6-dimethyl-phenylamino)-N,N-diethyl-N-(4-(methoxycarbonyl)benzyl)-2-oxoethanaminium bromide (1.0 g, 50 %). Mp: 183-185 °C. δ_H (500 MHz; DMF-D7) 1.61-1.64 (6H, t, *J* 7.2 Hz, 2 x CH_2CH_3); 2.31 (6H, s, 2 x Ar-CH_3); 2.73-2.78; 2.90-2.95 (CH_3, DMF-D7); 3.53 (H_2O in DMF); 3.74-3.82 (4H, m, 2 x CH_2CH_3); 3.96 (3H, s, O-CH_3); 4.72 (2H, s, N^+CH_2); 5.16 (2H, s, N^+CH_2); 7.12-7.17 (3H, m, 3 x Ar-*H*); 7.97-7.98 (2H, d, *J* 8.34 Hz, 2 x Ar-*H*); 8.03 (C*H*, DMF); 8.10-8.11 (2H, d, *J* 8.36 Hz, 2 x Ar-*H*); 10.88 (1H, s, N*H*). δ_C (500 MHz; DMF-D7; DEPT) 8.83 (CH_2CH_3); 18.94 (Ar-CH_3); 29.76-30.76; 34.89-35.89 (CH_3, DMF-D7); 52.89 (O-CH_3); 55.91 (CH_2CH_3); 57.19 (N^+CH_2); 62.22 (N^+CH_2); 127.94 (*C*H); 128.74 (*C*H); 130.48 (*C*H); 132.40 (*C*); 133.80 (*C*); 134.51 (*C*H); 134.75 (*C*); 136.23 (*C*); 162.62-162.09 (*C*H, DMF-D7); 163.55 (*C*=O); 166.73 (*C*=O). MS (ESI): m/z = 383.234 Da $(C_{23}H_{31}N_2O_3)^+$. Elemental analysis: calculated C 59.61 %; H 6.74 %; N 6.05 %; Br 17.24 %; found C 59.31 %; H 6.55 %; N 5.83 %; Br 16.5 %. IR (ATR): 3122, 2977, 2948, 2884, 1713, 1685, 1541, 1474, 1425, 1277, 1108, 761 cm^{-1} (**Supplementary Figure 5**)

Synthesis of compound 2

Lidocaine (1 g, 4.3 mmol) and methyl 3-(bromomethyl)benzoate (0.98 g, 4.3 mmol) were mixed at room temperature. After 24 hours THF (10 mL) was added and the mixture was stirred until the raw product converted into a white powder. The product was filtered off, washed two times with THF and dried at 40 °C to afford 2-(2,6-dimethyl-phenylamino)-N,N-diethyl-N-(3-(methoxycarbonyl)benzyl)-2-oxoethanaminium bromide (1.6 g, 80 %). Mp: 191-193 °C. δ_H (500 MHz; DMF-D7) 1.61-1.64 (6H, t, *J* 7.2 Hz, 2 x CH_2CH_3); 2.32 (6H, s, 2 x Ar-CH_3); 2.73-2.78; 2.91-2.95 (CH_3, DMF-D7); 3.52 (H_2O in DMF); 3.71-3.81 (4H, dq, *J1* 7.1 Hz, *J2* 13.55 Hz, 2 x CH_2CH_3); 3.94 (3H, s, O-CH_3); 4.68 (2H, s, N^+CH_2); 5.20 (2H, s, N^+CH_2); 7.13-7.16 (3H, m, 3 x Ar-*H*); 7.72-7.75 (1H, t, *J* 7.7 Hz, Ar-*H*); 8.03 (C*H*, DMF); 8.08-8.10 (1H, m, Ar-*H*); 8.16-8.18 (1H, m, Ar-*H*); 8.38 (1H, s, Ar-*H*); 10.87 (1H, s, N*H*). δ_C (500 MHz; DMF-D7; DEPT) 8.54 (CH_2CH_3); 18.77 (Ar-CH_3); 29.59-30.60; 34.73-35.73 (CH_3, DMF-D7); 52.69 (O-CH_3); 55.39 (CH_2CH_3); 56.66 (N^+CH_2); 62.12 (N^+CH_2); 127.80 (*C*H); 128.60 (*C*H); 129.46 (*C*); 130.26 (*C*H); 131.52 (*C*); 131.75 (*C*H); 134.55 (*C*H); 134.62 (*C*); 136.08 (*C*); 138.56 (*C*H); 162.60-162.92 (*C*H, DMF-D7); 163.45 (*C*=O); 166.55 (*C*=O). MS (ESI): m/z = 383.233 Da $(C_{23}H_{31}N_2O_3)^+$. Elemental analysis: calculated C 59.61 %; H 6.74 %; N 6.05 %; Br 17.24 %; found C 59.19 %; H 6.58 %; N 5.79 %; Br 12.3 %. IR (ATR): 3114, 2979, 2953, 2917, 1716, 1683, 1539, 1427, 1293, 1210, 1088, 757 cm^{-1} (**Supplementary Figure 6**).

Synthesis of compound 3

2-(2,6-dimethylphenylamino)-N,N-diethyl-N-(3-(methoxycarbonyl)benzyl)-2-oxoethanaminium bromide (**2**) (1 g, 2.2 mmol) was suspended in 50 mL ethanol and an excess of 0.05 M barium hydroxide (40 mL; 4 mmol) was added. The mixture was refluxed for 3 hours. After cooling to room temperature, 0.05 M sulfuric acid (40 mL; 4 mmol) was added. The precipitate was filtered off and the solution was evaporated to dryness. The precipitated white powder was washed two times with THF and dried at 40 °C to afford N-(3-carboxybenzyl)-2-(2,6-dimethylphenylamino)-N,N-diethyl-2-oxoethanaminium bromide (0.89 g, 90 %). Mp: 168-171 °C. δ_H (500 MHz; D_2O+NaOD) 1.52-1.55 (6H, t, *J* 7.12 Hz, 2 x $CH_2C\mathit{H}_3$); 2.13 (6H, s, 2 x Ar-$C\mathit{H}_3$); 3.44-3.60 (4H, qdd, *J1* 6.85 Hz, *J2* 13.98 Hz, *J3* 44.92 Hz, 2 x $C\mathit{H}_2CH_3$); 4.89 (2H, m, $N^+C\mathit{H}_2$ + D_2O); 4.91 (2H, s, $N^+C\mathit{H}_2$); 6.94-6.97 (1H, t, *J* 7.47 Hz, Ar-*H*); 7.09-7.11 (2H, d, *J* 7.45 Hz, 2 x Ar-*H*); 7.59-7.62 (1H, t, *J* 7.7 Hz, Ar-*H*); 7.80-7.82 (2H, d, *J* 7.68 Hz, 2 x Ar-*H*); 8.00-8.01 (2H, d, *J* 7.82 Hz, 2 x Ar-*H*); 8.06 (1H, s, Ar-*H*). δ_C (500 MHz; D_2O+NaOD; DEPT) 10.43 ($CH_2\mathit{C}H_3$); 20.58 (Ar-$\mathit{C}H_3$); 56.38 ($\mathit{C}H_2CH_3$); 63.57 ($N^+\mathit{C}H_2$); 70.68 ($N^+\mathit{C}H_2$); 126.56 (*C*H); 130.34 (*C*H); 130.51 (*C*); 131.94 (*C*H); 133.49 (*C*H); 133.88 (*C*); 135.70 (*C*H); 138.18 (*C*H); 140.00 (*C*); 149.15 (*C*); 163.75 (*C*=O); 177.55 (*C*=O). MS (ESI): m/z = 369.217 Da $(C_{22}H_{29}N_2O_3)^+$. Elemental analysis: calculated C 58.80 %; H 6.50 %; N 6.23 %; Br 17.78 %; found C 56.50 %; H 6.47 %; N 5.84 %; Br 14.9 %. IR (ATR): 3526, 3453, 3083, 3003, 2980, 1705, 1678, 1520, 1470, 1442, 1370, 1235, 1184, 779 cm^{-1} (**Supplementary Figure 7**).

Synthesis of denatonium derivatives coupled to aminoacids

Synthesis of compound 4

Lidocaine (0.3 g, 1.3 mmol) and methyl 2-(4-(bromomethyl)phenyl)acetate (0.3 g, 1.2 mmol) were mixed at room temperature. After 24 hours THF (5 mL) was added and the mixture was stirred until the raw product converted into a white powder. The product was filtered off, washed two times with THF and dried at 40 °C to afford 2-(2,6-dimethylphenylamino)-N,N-diethyl-N-(4-(2-methoxy-2-oxoethyl)benzyl)-2-oxoethanaminium bromide (0.33 g, 55 %). Mp: 93-103 °C. δH (500 MHz; DMF-D7) 1.59 (6H, m, 2 x CH2CH3); 2.32 (6H, s, 2 x Ar-CH3); 2.74; 2.91 (CH3, DMF-D7); 3.69 (6H, m, 2 x CH2CH3 + Ar-CH2); 3.84 (3H, s, O-CH3); 4.70 (2H, s, N+CH2); 5.06 (2H, s, N+CH2); 7.14 (3H, m, 3 x Ar-H); 7.49-7.50 (2H, m, 2 x Ar-H); 7.76-7.77 (2H, m, 2 x Ar-H); 8.05 (CH, DMF); 10.99 (1H, s, NH). δC (500 MHz; DMF-D7; DEPT) 8.82 (CH2CH3); 18.95 (Ar-CH3); 29.70-30.71; 34.86-35.86 (CH3, DMF-D7); 40.54 (Ar-CH2); 52.26 (O-CH3); 55.43 (CH2CH3); 56.87 (N+CH2); 62.52 (N+CH2); 127.24 (C); 127.83 (CH); 128.63 (CH); 130.84 (CH); 133.91 (CH); 134.65 (C); 136.09 (C); 137.89 (C); 162.53-163.00 (CH, DMF-D7); 163.41 (C=O); 172.09 (C=O). MS (ESI): m/z = 397.249 Da (C24H33N2O3)+. Elemental analysis: calculated C 60.38 %; H 6.97 %; N 5.87 %; Br 16.74 %; found C 59.48 %; H 7.01 %; N 5.30 %; Br 15.2 %. IR (ATR): 3119, 2974, 2951, 2914, 1731, 1679, 1537, 1437, 1229, 1155, 781 cm-1. **(Supplementary Figure 8)**

Synthesis of compound 5

4-(bromomethyl)benzoic acid (1 g, 4.65 mmol), glycine ethyl ester hydrochloride (0.65 g, 4.65 mmol), dicyclohexyl carbodiimide (0.96 g, 4.65 mmol) and dimethylaminopyridine (0.57 g, 4.65 mmol) were dissolved in 200 mL of methylene chloride. The mixture was stirred at room temperature for about 16 h. After filtering and washing (methylene chloride) of the precipitated dicyclohexylurea, the solution was concentrated to a crude product which was purified via column chromatography (silica gel 60, methanol/chloroform 1:10 v/v) and dried at 40 °C to afford ethyl 2-(4-(bromo methyl)benzamido)acetate (1.2 g, 86 %).

Lidocaine (0.39 g, 1.67 mmol) and ethyl 2-(4-(bromomethyl)benzamido)acetate (0.5 g, 1.67 mmol) were mixed at room temperature. The mixture was heated for 1 hour at 80 °C. After 24 hours THF (5 mL) was added and the mixture was stirred until the raw product converted into a white powder. The product was filtered off, washed two times with THF and dried at 40 °C to afford 2-(2,6-dimethylphenylamino)-N-(4-(2-ethoxy-2-oxoethylcarbamoyl)benzyl)-N,N-diethyl-2-oxoethanaminium bromide (0.40 g, 45 %). Mp: 174-176 °C. δH (500 MHz; D2O) 1.31-1.34 (3H, t, J 7.15 Hz, OCH2CH3); 1.53-1.56 (6H, t, J 7.22 Hz, 2 x CH2CH3); 2.25 (6H, s, 2 x Ar-CH3); 3.56-3,68 (6H, m, 2 x CH2CH3); 4.22 (4H, s, NH-CH2$^+$ N$^+$CH2); 4.27-4.31 (2H, q, J 7.15 Hz, OCH2CH3); 4.80 (D2O); 4.89 (2H, s, N$^+$CH2); 7.20-7.28 (3H, td, J1 6.90 Hz, J2 22.20 Hz, 3 x Ar-H); 7.68-7.69 (2H, d, J 8.36 Hz, 2 x Ar-H); 7.94-7.96 (2H, d, J 8.36 Hz, 2 x Ar-H). δC (500 MHz; D2O; DEPT) 8.40 (CH2CH3); 14.38 (OCH2CH3); 18.42 (Ar-CH3); 42.94 (OCH2CH3); 55.69 (CH2CH3); 56.35 (N$^+$CH2); 62.31 (NH-CH2); 63.59 (N$^+$CH2); 129.08 (CH); 129.37 (CH); 129.49 (CH); 131.86 (C); 132.98 (C); 134.11 (CH); 136.02 (C); 136.74 (C); 164.70 (C=O); 170.73 (C=O); 172.60 (C=O). MS (ESI): m/z = 454.270 Da (C26H36N3O4)$^+$. Elemental analysis: calculated C 58.43 %; H 6.79 %; N 7.86 %; Br 14.95 %; found C 61.84 %; H 7.24 %; N 8.22 %; Br 2.95 %. IR (ATR): 3246, 3124, 2980, 2963, 2931, 1750, 1678, 1656, 1540, 1470, 1373, 1204, 766 cm-1 (**Supplementary Figure 9**).

EDC-NHS to form the acetylated Diaminoethyl Denatonium

50 mM of EDC was added to 5 mM of 3 in water (pH of 4.4 – 4.8). 200 mM of Sulfo-NHS was added to the reaction (pH 5 – 5.3). The reaction components were mixed and reacted for 45 minutes at room temperature. Afterwards N-(2-aminoethyl)acetamide in a 10-fold molar excess to N-(3-carboxybenzyl)-2-(2,6-dimethylphenylamino)-N,N-diethyl-2-oxoethanaminium bromide was added. The reaction was allowed to proceed for 4-6 h at RT. The product Diaminoethyl Denatonium acetylated ((Diaminoethyl-D., (Mw = 453 Da)) was analyzed at a LC-MS-system and purified using FPLC (**Supplementary Figure 10**)

Nuclear magnetic resonance and Infrared spectroscopy

1H Nuclear magnetic resonance (NMR) and 13C NMR spectra were measured on a Varian Inov 500 MHz spectrometer (deutero, Kastellaun, Germany). The differentiation of primary, secondary and tertiary carbon atoms was realized by DEPT (Distortionless enhancement by polarization transfer)

experiment. Infrared spectroscopy (IR)-spectra were obtained on a FT-IR-Spectrometer Nicolet Impact 410 (Thermo Scientific, Waltham, MA) applying ATR (attenuated total reflection) technique.

Peptide synthesis

All PCL sequences and other peptides were manually synthesized in-house using Fmoc acid – SPPS and different resins. Multiple batch-batch variations were provided. For peptides 1, 1a, 1b, 1c, 3 4 and 6 (**Supplementary Figure 11, Supplementary Table 1**), Fmoc-Gln(Trt)-Wang or Fmoc-Gly-Wang resin was loaded into in a polypropylene-reactor (MultiSynTech, Witten, Germany). For peptides 5, 5a, and 7 (**Supplementary Figure 11, Supplementary Table 1**) Fmoc-Rink-Amid PEG AM Resin, Fmoc-Rink-Amid-AM and Fmoc-Rink-Amid-PS-AM and for peptides 2, 2a and 2b (**Supplementary Figure 11, Supplementary Table 1**) CTC-resin was used, respectively. For the CTC-resin, coupling of the first amino acid was with a 5-molar excess of the respective first Fmoc-protected amino-acid (excess was in comparison to the loading of the resin) with 0.2 M DIPEA in DCM and incubated for at least 1h. Subsequently, free bindings sites were blocked with pure methanol for 15 minutes and washed using DCM (3x), DMF (2x), DCM (2x,) and methanol (3x). For coupling of the subsequent amino acids, the respective amino acid was dissolved in 2.5 mL of 0.4 M HBTU (coupling agent) in DMF and 250 µL DIPEA were added. The resulting solution was used in 5 molar excess with respect to the amino acid as compared to the loading of the resin (for His, Cys, or Trp, 157 µL instead of 250 µL DIPEA was added). Removal of the protecting Fmoc group was with 40 % piperidine in DMF for 3 minutes and subsequently with 20 % piperidine in DMF for 10 minutes. For all other resins than CTC (Fmoc-Gln(Trt)-Wang resin, Fmoc-Gly-Wang, Fmoc-Rink-Amid-PEG-AM resin, Fmoc-Rink-Amid-AM, and Fmoc-Rink-Amid-PS-AM), the respective coupling step was with 250 µL of the amino acid solution (5-molar excess of the respective amino acids in comparison to the loading of the resin) in 0.5 M HOBt in DMF, followed by 80 µL DIC and 88 µL DIPEA. The azido group was introduced by a 2-molar excess (2-molar excess of the respective amino acids in comparison to the loading of the resin) of Fmoc-β-azido-Ala-OH (Mw = 352.34 Da) or Fmoc-L-Dap(N_3)-OH (Mw = 366.41 Da). N-terminal fluorescence labeling was by adding 5,6-carboxyfluorescein (5-molar excess in comparison to the loading of the resin) following the standard SPPS protocol.

N-terminal denatonium labeling (carboxyl functionalized denatonium (referred to as carboxy-denatonium, i.e. N-(3-carboxybenzyl)-2-(2,6-dimethylphenylamino)-N,N-diethyl-2-oxoethanaminium bromide; synthesis *vide infra*) to the peptide on the resin was with 37.5 mg carboxy-denatonium and 38.28 mg HOBt in 500 µL of a 1:4 solution of H_2O/DMF (v/v). This mixture was transferred to the peptidyl resin, 20 µL of DIC were added and the reactor was shaken for at least one hour.

Selective removal of the Mtt group protecting lysine's ε-amino group (Mtt group protected; Fmoc-Lys(Mtt)-OH) was with 2 cycles of 2 % TFA and 2.5 % TIS in DCM each for 10 minutes. Afterwards, the resin was neutralized by washing with 10 % DIPEA in DCM.

Final peptides cleavage from the resins was with 95 % TFA and 5 % of the scavenger mixture - ethanedithiol, m-cresol, thioanisole, water (1:2:2:2, (v/v)) - for 3 hours followed by precipitation with

diethyl ether at -20 °C. The suspension was centrifuged for 5 minutes (5.500 1/min; Sigma 3K12 centrifuge), the supernatant was discarded and washed two times with diethyl ether. The resulting precipitate was dissolved in 3 % acetonitrile, 0.1 % TFA in water (added *ad libitum* until completely dissolved).

Peptide esterfication (Steglich esterification)

For peptide esterification, DCC was used as coupling reagent and DMAP as catalyst. Freeze dried peptides were dissolved in water-free methanol, then 5 eq DMAP (with respect to the peptide) and 1 eq DCC were added. The reaction was allowed for 5 days at room temperature and monitored by HPLC (method, *vide infra*).

Peptide acetylation

Acetylation of peptide #7 **(Supplementary Table 1)** was performed in a twostep reaction. First the resin-bound peptide was incubated with 100 µL acetic anhydrite and 90 µL DIPEA in 2 mL DMF for 30 min with the aim of acetylating the N-terminal amino group. After cleavage from the resin the peptide, carrying a C-terminal amide, was dissolved in 2 mL DMF with 200 µL acetic anhydrite and 90 µL DIPEA and incubated at room temperature overnight followed by precipitation with diethyl ether at -20 °C.

Matrix-assisted laser desorption/ionization (MALDI)

For MALDI analysis, 0.5 µL of the peptide synthesis product was embedded in a matrix consisting of equal parts of α-cyano-4-hydroxycinnamic acid in a 6:4 (v/v) dissolved in 60 % acetonitrile with 0.3 % TFA in water. MALDI-MS spectra were acquired in the linear positive mode by using an Autoflex II LRF instrument from Bruker Daltonics (Billerica, MA). Mass spectra were calibrated externally with a peptide calibration standard (Bruker Daltonics).

Analytical high-performance liquid chromatography (HPLC)

Analytical HPLC was carried out on a Hitachi Elite LaChrom HPLC System (VWR, Darmstadt, Germany) with a ZORBAX Eclipse XDB-C18 column (4.6 mm internal diameter, 150 mm length (Agilent, Santa Clara, CA)), with eluent A (0.1 % TFA in water, (v/v)) and eluent B (0.1 % TFA in 2:3 water-acetonitrile (v/v)) and a gradient from 1 to 60 % eluent B within 55 minutes. UV absorption was read at $\lambda = 220$ nm. HPLC-spectra were used for determination of peptide purity (e.g. **Supplementary Table 1, Supplementary Figure 11**) and analysis of PCL cleavage (e.g. **Figure 2, 3**) by analysis of the peak area of the main peak.

Preparative peptide purification

Preparative purification was carried out by fast protein liquid chromatography FPLC on a GE ÄKTA Explorer (GE Healthcare, Chalfont St Giles, UK) system with a Jupiter 15u C18 300 A column (21.2 mm internal diameter, 250 mm length (Phenomenex Inc., Torrance, CA)), with eluent A (0.1 % TFA

in water (v/v)) and eluent B (0.1 % TFA in 2:3 water-acetonitrile (v/v)) and a gradient from 33 to 80 % eluent B within 30 minutes. UV absorption was read at λ = 280 nm or 220 nm. After freezing the samples at -80 °C, freeze-drying was performed on a Christ Alpha 1-4 device (Martin Christ Gefriertrocknungsanlagen, Osterode am Harz, Germany) at a desublimers' temperature of -60 °C, pressure of 1.030 mbar and processing temperature of 19 °C.

MMP digestion

Pro-MMPs (e.g. MMP-8, MMP-1 or MMP-9; adapted to 0.1 mg/mL and > 100.0 mU/mg based on manufacturer information and when appropriate) were activated with APMA [8]. For that, a 10 mM stock solution of APMA in 0.1 M NaOH was freshly prepared and a 10:1 (v/v) ratio of the respective proenzyme and APMA solution (MMP:APMA) was incubated for 3 h at 37 °C. The activity was confirmed using the MMP-8 detection kit (QuickZyme® Biosciences, Leiden, Netherlands). A stock solution (90 µg/mL) of the activated MMP was prepared (used for cleavage studies on the protease-cleavable linker (PCL; e.g. **Figure 2**) or the fully assembled microparticle-PCL-tastant setup (sensor; e.g. **Figure 3**), in MMP-8 buffer (200 mM NaCl, 50 mM Tris-HCl, 5 mM $CaCl_2$, 1 µM $ZnCl_2$, 0.05 % Brij35 at pH 6.8-7.0). All MMP-8 experiments were performed as follows. 25 µg of the sensor (see chapter: Peptide-coupling to beads (Sensor)) in 100 µL MMP-buffer or 1 mg of FPLC-purified and lyophilized PCL as described dissolved 2 mL MMP-buffer resulting in a final concentration of 0.5 mg/mL substrate was used.

For the analysis of the impact of MMP-8 concentration, the protease was added in concentrations of 0, 9, 45, 90, 225, 450, 900 ng/mL, respectively, and incubated at 37 °C under agitation for 1 hour (e.g. **Figure 2*A*, 3, 4*B***). For the analysis of the impact of MMP-8 incubation time, a MMP-8 concentration of 900 ng/mL was used under otherwise identical conditions and incubated for 1, 2, 3 and 6 hours. For the analysis of MMP selectivity, MMP-1 (56 kDa), MMP-9 (92 kDa) and MMP-13 (52 kDa) were firstly activated following identical procedures as outlined above for MMP-8 (excluding MMP-13 which was obtained in activated from) and at a concentration of 900 ng/mL (e.g. **Figure 3, 4*A***).

Enzymatic reactions were stopped using 4 µL 250 mM EDTA in case of the sensor or heating in case of uncoupled PCL (95 °C for 15 minutes; the beads of the sensor would melt at heating due to a different method used). Cleavage was analyzed by MALDI-MS analysis (PCL) or by flow cytometry (sensor) in comparison to negative controls (the negative controls for the PCL and sensor experiments were by incubation without MMP under otherwise identical conditions). The relative decrease of the PCL's main peak was characterized by HPLC (PCL) or of the sensor's fluorescence by FACS (sensor) in comparison to control. Resulting fragments were characterized by MALDI-MS or LC-MS for cleavage experiments on the PCLs but not for the sensor (**Supplementary Figure 12, 13*B***).

MMP-activity determination

Determination of MMP-activity of self-activated MMPs was conducted using the human MMP-8 activity assay (QuickZyme® , Leiden, The Netherlands). APMA activated MMPs (MMP-1, MMP-8,

MMP-9) in a concentration of 0.4 ng/µL (50 µL) were mixed with MMP-8 buffer (37 µL), detection enzyme (5 µL) and substrate (8µL; all from QuickZyme®). The activity was measured for 12 hours in intervals of 10 minutes. The reaction rate was calculated from the slope from the initial linear period.

Aminopeptidase (AP) incubation

Leucine Aminopeptidase (AP; Mw = 280 kDa) from porcine kidney dissolved in AP buffer (50 mM Tris-HCl, 1 mM $CaCl_2$ and 150 mM NaCl at pH 7.0) was used. L-leucine-p-nitroanilide (0.125 mg/mL) was used as control substrate. For AP digestion, 1 mg of purified lyophilized peptide was diluted into 2 mL AP-buffer resulting into a final concentration of 0.5 mg/mL substrate. To evaluate the impact of AP concentration on peptide cleavage, AP was added at concentrations of 0 (negative control), 0.87, 8.7, 39, and 78 µg/mL and the samples were shaken at 37 °C for 1 hour. For analysis of the impact of AP incubation time, the identical conditions were used (AP concentration of 0.87 µg/mL (and analyzed after 10, 20 minutes and 1, 2, 3 and 6 hours). To stop the enzymatic activity, samples were kept at 95 °C for 15 minutes. Afterwards, HPLC analyses were performed (as described) to analyze the relative decrease of the main peak as compared to the negative control (see **Figure 2*B*, *C***).

The entire degradation pattern of the first generation *versus* second generation PCL was studied (**Supplementary Figure 13*A*-*G***). For that, first generation and second generation peptides were first exposed to 1800 ng/mL to MMP for 22 h (for conditions, see above), followed by exposure to AP at concentrations of 0.87 µg/mL for 30 minutes at 37 °C (for conditions, see above). The complete degradation of the resulting second generation peptide (MMP-8 cleavage fragment (IAGQK-De (ID5a) – manually synthesized)) was analyzed by exposure to AP (87 µg/mL) for 20 h at 37 °C (**Supplementary Figure 13 *F*,*G* -** for conditions, see above).

For AP-incubation analysis a Shimadzu Corp. (Kyōto, Japan) LC-MS-system, containing a DGU-20A3R degassing unit, a LC20AB liquid chromatograph and a SPD-20A UV/Vis detector was used. Mass spectra were obtained by a LC-MS 2020 (Shimadzu Corp.). A Synergi 4U fusion-RP (4.6 mm internal diameter, 150 mm length) column (Phenomenex) with eluent A (0.1 % formic acid in water (v/v)) and eluent B (0.1 % TFA in MeOH + (v/v)) was used. Parameters for method: A: (water +), B: V(B)/(V(A)+V(B)) = 5 % to 90 % over 10 min, V(B)/(V(A)+V(B)) = 90 % for 5 min, V(B)/(V(A)+V(B)) = 90 % to 5 % over 3 min. The method was performed with a flow rate of 1.0 mL/min. UV detection was measured at 254 nm.

For determination of AP-specificity the first generation PCLs D.-CLAN$_3$Q (ID #1c) and AN$_3$CL-D. (ID #5) were compared. After 22 h incubation with 1800 ng/mL MMP-8 both PCLs were completely cleaved. An additional AP-incubation (3.1 nM) for 30 minutes results in no difference for PCL D.-CLAN$_3$Q (ID #1c) and emerging peaks for AN$_3$CL-D. (ID #5) (**Supplementary Figure 13**). This confirmed an already described N-terminal hydrolysis activity of AP [9]. The second generation peptides (either the resulting fragment after PCL digest with MMP-8, or the manually synthesized fragment (IAGQK-De (ID #5a); Mw = 865.65 Da)) was incubated with AP (for conditions, see above). Mass

detection confirms hydrolysis of this fragment resulting in lysine bound denatonium (Mw = 497.4 Da; (**Supplementary Figure 13*D,G***).

For the determination of salivary AP-concentration patients in a private dental surgery were defined as having either an inflammation in the oral cavity (advanced periodontitis and odontogenic infection) or as healthy patients. The anonymized study was approved by the ethics committee at the faculty of medicine of the University of Wurzburg.

The AP-specific substrate l-leucine-p-nitroanilide (0.5 mg/mL) in AP-buffer was used for determine the AP-concentration in saliva samples. Linearity was determined by serially diluting AP from porcine kidney dissolved in AP buffer and comparing observed values with expected values. After an incubation time of 30 min a microplate reader (Tecan SpectraMax250, Molecular Devices, CA, USA) at a wavelength of 405 nm was used for determination (**Supplementary Figure 14)**. The concentration for each salivary sample was calculated from the standard curve using linear regression analysis.

Salivary samples were taken from different peoples and a three-fold determination was performed.

Peptide-coupling to beads (Sensor)

For the Cu(I) catalyzed cycloaddition reaction (click reaction), 5,6-carboxyfluoresein coupled azido-peptides and custom-made, transparent PMMA-beads carrying alkyne functional groups were used.

For this project the alkyne functional groups were covalently coupled on the surface of the beads using a photoinitiated grafting procedure by the use of propargylacrylamide as monomer and bezophenone as photoinitiator. The results are core-shell beads with a PMMA core and a shell of poly-propargylamide with accessible propargyl groups for further click chemistry.

Alkyne-beads (1-2 mg) were coupled to the PCL in 100 µL Tris-buffer (50 mM, pH 7.5), 2 µL of a 1:1 (v/v) mixture of 100 mM Cu_2SO_4 and 100 mM TCEP in water, 0.4 µL TBTA (5 mM in DMSO, tertiary butanol (1:4, v/v)), and 10 µL azido-peptide (4 mM) in aluminium foil wrapped (light protected) Eppendorf tubes. After incubation for 60 minutes, the beads were washed by addition of 500 µL water for 10 minutes, centrifuged and the supernatant was discarded. The remaining particles were washed in 500 µL EDTA in water (0.1 %, m/v) for 10 minutes, centrifuged, and the supernatant was discarded. This washing procedure was repeated two additional times using 500 µL sodium laurylsulfate (SDS) in water (1 %, m/v) for 10 minutes each and again two times with 500 µL water for 10 sec. Negative controls were prepared under identical reaction conditions without Cu_2SO_4 and TCEP. Finally, the beads were resuspended in 500 µL water (LCMS grade) and analyzed by fluorescence microscopy and flow cytometry.

Fluorescence microscopy

Azido-alkyne coupling of the carboxyfluorescein-modified PCLs to PMMA-particles was analyzed using an inverted epifluorescence microscope Axio Observer Z1 (Zeiss, Oberkochen, Germany) with a

Plan-Apochromat 40x/0.95 objective (Zeiss, Oberkochen, Germany) and fluorescence excitation at λ = 450-490 nm, dichroic beam splitter at λ = 495 nm, emission bandpass at λ = 500-550 nm and an exposure time of 1 second.

Flow cytometric analysis (FACS)

At least 15000 particles were analyzed by flow cytometry (FACSCalibur, Becton Dickinson, Franklin Lakes, NY) by recording side scatter, forward scatter, and fluorescence signals using an argon-ion laser line at λ = 488 nm for excitation and an optical filter centered on $\lambda = 530 \pm 30$ nm and a pass-band of 30 nm and evaluated by BD cellquest pro software. The level of non-specific binding was determined using control PMMA particles (prepared in absence of Cu_2SO_4 and TCEP).

Data were analyzed in density plots. Each dot represents an individual particle that has passed through the instrument. The light scatter signals of PMMA-particles were distinguished from unspecific fluorescent particles by gating (**Supplementary Figure 15**).

Electronic tongue measurements

Taste evaluation was carried out with the potentiometric electronic taste sensing system TS-5000Z (Insent, Atsugi-Shi, Japan). Taste sensing systems, also called electronic tongues, are analytical sensor array systems, which are able to detect specific substances by means of different artificial membranes and electrochemical techniques. In principle, these systems “try to represent and imitate what is happening while molecules with specific taste properties interact with taste buds on the human tongue” [10]. In connection with the applied instrument, which works on a potentiometric basis, eight sensors represent the human taste buds, whereas sensors: SB2AC0 and SB2AN0 are dedicated to bitter cationic substances, sensor SB2AAE is dedicated to umami, sensor SB2CT0 is dedicated to saltiness, sensor SB2CA0 dedicated to sourness, sensor SB2C00 is dedicated to bitter anionic substances and sensor SB2AE1 is dedicated to astringent substances. Once surface of these sensors interact with according molecules, changes in the electric potential can be detected. Good correlation between electronic tongue data and human sensory panel data was presented for charged molecules of differing chemical character by several working groups [11, 12].

To prove proper sensor performance, a so called sensor check is performed prior each measurement. Each sensor is therefore dipped into a solution with a defined ionic content and strength for 30 seconds, until the difference in sensor signals is < 0.5 mV between the last measurements. To avoid sensor saturation and therewith falsified sensor signals during sample measurement, the sample sequence is measured four times instead of measuring each sample four times in a row. The first out of the four measurements was discarded for further calculations to avoid any unstable data. Hence, the presented values are the arithmetic mean of three subsequent measurements. Sensor responses were recorded in mV. For a more detailed description of electronic tongue measurement, please see also [13, 14, 15]. Each sample was measured four times and the first measurement was discarded for further

calculations to avoid any unstable data. Hence, the presented values are the arithmetic mean of three subsequent measurements. Sensor responses were recorded in mV.

Dependent on the incorporated artificial lipids in the sensor membranes, sensors should be dipped within the washing procedure into either the (-)- or the (+)-washing solution. The (-)-washing solution was prepared by diluting 100 mM hydrochloric acid (Merck, Darmstadt, Germany) with ethanol (30 % (w/w)), the (+)-washing solution by dissolving 100 mM potassium chloride and 10 mM potassium hydroxide in ethanol (30 % (w/w)). The standard solution, which served as cleaning and reference solution, was prepared by dissolving 0.3 mM tartaric acid and 30 mM potassium chloride in distilled water.

Data were evaluated uni- and multivariate. For univariate evaluation, Excel 2010 (Microsoft, Redmond, US) was used. Multivariate statistics were performed by using SIMCA-P 13.0 (Umetrics AB, Umea, Sweden).

Sensor signals of the potentiometric electronic tongue can increase or decrease or do not change with respect to changing concentrations of the investigated substance. As a successful bitterness reduction is directly correlated with less detected tastant, most valid results will be obtained with tastants showing concentration-dependent sensor signals. As the relationship between sensor response and tastant concentration is substance-specific, it is thus mandatory to investigate dilution series of each substance of interest. Therefore, all substances were measured in different concentrations with all sensors. To furthermore get comparable results, each substance was evaluated in relation to denatonium.

Within the univariate evaluation, concentration dependent sensor signals as summarized in **Supplementary Table 2** were found. Univariate evaluation revealed that Denatonium methyl ester (**Supplementary Figure 6**) and Denatonium $(G)_1$ ester (**Supplementary Figure 9**) are comparably detected as Denatonium, in particular by those e-tongue sensors, which are dedicated to cationic bitterness (SB2AN0, SB2AC0). Measurement of Denatonium $(G)_4$ ester (ID #6) and K-Denatonium acetylated (ID #7) concentration series resulted in less sensitive cationic bitter sensor responses, but still concentration dependent signals of the astringency sensor (SB2AE1) and the sensor dedicated for anionic bitterness (SB2C00). As Denatonium (G) ester contains a lot more charges, e-tongue measurement might have, however, not led to appropriate results regarding the bitterness evaluation.

A more distinctive view on the overall e-tongue results can be provided by multivariate evaluation, including the information of all bitter sensors (SB2AC0 SB2AN0 bitter cationic and SB2C00 bitter anionic). **Supplementary Figure 16** shows the Scores Scatter Plot of an according principal component analysis (PCA). For an appropriate interpretation of this PCA Scores Scatter Plot, the distances of the data points, the percentages on the x- and y-axes (**Supplementary Figure 16**) and the according Loading Scatter Plot (**Supplementary Figure 17**) have to be considered.

Regarding the distance: the closer the data points of two samples are, the more similar they have been detected by the sensors which indicates a similar taste; regarding the percentage: **Supplementary Figure 16** displays that 91.7 % of the information is given by the sample distribution along the x-axis, representing most of the data, while 4.3 % of the information are distributed along the y-axis. The Loading Scatter Plot (**Supplementary Figure 17)** shows that data points are predominantly directed rightwards in the Scores Scatter Plot by the sensors SB2AN0 and SB2AC0, which are dedicated to bitterness of cationic substances, reflecting the predominant charging of the investigated molecules. If the different concentrations of the bitter model Denatonium and moreover the location of the non-bitter sample (buffer) are considered (**Supplementary Figure 16**), it can be stated that the samples get the more bitter the further they are located at the right side of the map and each sample is bitterer than the buffer sample. Taking this evaluation into account, it can be assumed that bitterness is reduced according the following sequence: Denatonium (0.5 mM) > Denatonium methyl ester (0.5 mM) > Denatonium $(G)_1$ ester (0.5 mM) > Denatonium (0.1 mM) > Diaminoethyl Denatonium acetylated (0.5 mM) > K-Denatonium acetylated > Denatonium (0.05 mM).

Tableting of the chewing gum

Chewing gum and pressing

For the production of medicated chewing gum three directly compressible powder (PWD) (Health in Gum (HIG)® Basics CAFOSA GUM SAU, Barcelona, Spain) were used in combination with PMMA-particles differing in bead-size. HIG composition and manufacturer's suggested recipes are mentioned in **Table S3**.

The powder was mixed in a Turbula mixer (Bachhofen, Muttenz, Switzerland) and tableting was performed on an instrumented eccentric press (EK0, Korsch, Berlin, Germany). Powder characterization of the Health in Gum Basics and the manufacturer's suggested recipes include the study of angle of repose α, Hausner ratio, compressibility index (Carr index) and the powder flow rate and break force measurements were achieved according to compendial monographs [16]. In order to make a decision on the most appropriate basis for the production of chewing gum, all three formulations were compressed with the instrumented eccentric press EK0 (Korsch, Berlin, Germany). During the pressing process "PMA3 eccentric", the upper punch force-way and the upper punch force were recorded with the aid of the program. From the recorded data using an upper punch force-way diagram was created for each recipe. For better comparison, the individual curves were plotted on a graph (**Supplementary Figure 18**). Tableting of HIG PWD-03 resulted in mechanically stable pharmaceutical forms, with break forces exceeding 100 N sufficient for packaging and transport requirements.

Clinical protocol

For the collection of sulcus fluid and saliva from peri-implant diseased patients and from healthy volunteers, a cross-sectional study was initiated with the patient group having either a mucositis or a peri-implantitis *vs*. healthy control. Peri-implant diseased patients were defined as having either a mucositis or peri-implantitis (**Supplementary Figure 19**), reflecting the later use of the chewing gums to detect inflammation in the oral cavity and not to discriminate between mucositis or peri-implantitis (**Figure 6**). The study was approved by local authorities ('Servizio Sanitario Regionale Emilia-Romagna' Azienda Unità Sanitaria Locale di Rimini U.O. Qualità,Ricerca organizzativa e Innovazione Prot. n. 0134011 Rimini, 20/12/11) and was conducted according to the Declaration of Helsinki. In this comparative study, the ability of the sensor diagnostic was assessed to discriminate saliva collected from patients with peri-implant disease to asymptomatic carriers of at least one implant (referred to as healthy control). The performance of the sensor diagnostic was shown in parallel to the performance of two commercially available tests, one measuring from sulcus fluid samples (chair side test from Dentognostics), one measuring from saliva (the Quickzyme test). Sample sizes of the groups were chosen based on previous studies using the dental chair-side test from Dentognostics [17]. Patients were enrolled after giving their written consent. General inclusion criteria were an age above 18 years and all subjects had at least one titanium dental implant. General exclusion criteria were pregnancy or lactating period, patients with a history of systemic disorders, antibiotics/anti-inflammatory drug administration within the last 30 days, substance abusers, symptoms of acute illness (e.g., fever, sore throat, diarrhea), presence of an oral mucosal inflammatory condition (e.g., aphthous, lichen planus, oral injuries, leukoplakia and oral cancer). Sulcus fluid was collected from the peri-implant zone using standardized MMP-8 collection strips (5-GCF/PISF strips, Dentognostics GmbH, Jena, Germany). The operator took 4 samples from 4 different sites at each implant. These samples were sent to the service lab Dentognostics at which this commercially available approach for the analysis of sulcus fluid was conducted (referred to as chair side test) [18]. Additionally to the sulcus fluid sampling, a further saliva sampling was performed in the patients. Unstimulated saliva was collected using an absorbent device (SalivaBio Oral Swab (SOS) from Salimetrics, Carlsbad, CA).

Also the occurrence of periodontitis in the patients (periodontitis n = 20; no periodontitis n = 13) was reported and compared to the three test systems (**Supplementary Figure 20**).

The intention to treat (ITT) cohort had n = 40 patients with peri-implant disease (n = 20 for peri-implantitis and n = 20 for mucositis at the implant site) and n = 20 healthy volunteers (with no inflammation at the implant site). As of the low donation volume of saliva collected from individuals, we only analyzed data from samples with sufficient saliva volume allowing for all saliva tests, i.e. the protease activity test (see above for section MMP-activity determination) and for the two incubation times used for testing our sensor (see below for section Analysis of saliva from peri-implant disease patients and healthy volunteers). The examiners of the laboratories were blinded to group assignment.

Analysis of saliva

Determination of total MMP-activity in saliva-samples was conducted using the human MMP-8 activity assay (QuickZyme®, Leiden, The Netherlands) according to manufacturer instructions. Saliva samples from the cross-sectional (saliva from peri-implant disease patients and healthy volunteers) study were incubated 1:1 with 0.1 mg/mL (De-CLAN$_3$Q) (ID #1c) in MMP buffer (200 mM NaCl, 50 mM Tris-HCl, 5 mM $CaCl_2$, 1 µM $ZnCl_2$, 0.05 % Brij 35 at pH 6.8-7.0) at 37°C under agitation for 10 min as well as for 1 h. Pipetting was done on ice. Enzymatic reactions were stopped by heating (95 °C for 15 minutes). The relative decrease of the PCL's main peak was characterized by HPLC (PCL) in comparison to control.

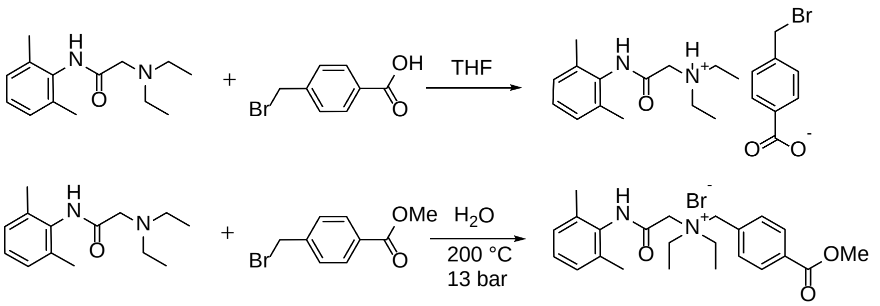

Supplementary Figure 1: Reaction of lidocaine with carboxylated benzyl bromides in solution.

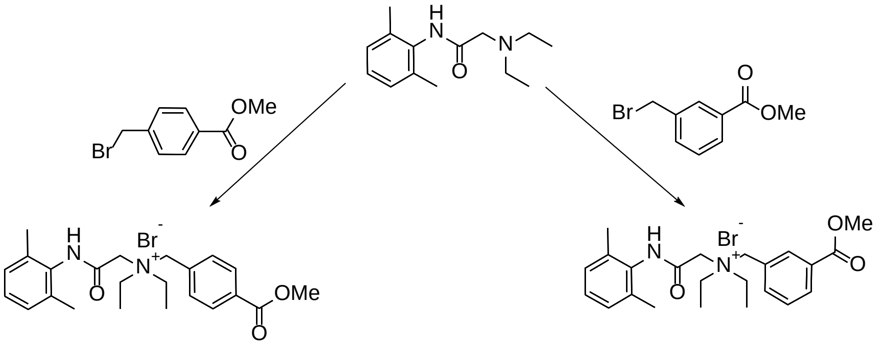

1 **2**

Supplementary Figure 2: Reaction of lidocaine with methyl 4- and 3-(bromomethyl)benzoate, respectively in solid state. The reaction of the two solids lidocaine and methyl bromomethylbenzoate starts shortly after mixing.

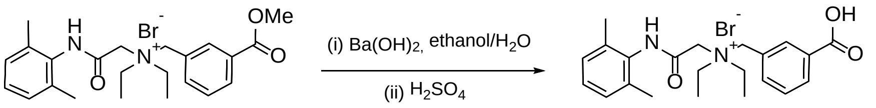

2 **3**

Supplementary Figure 3: Alkaline hydrolysis of compound 2.

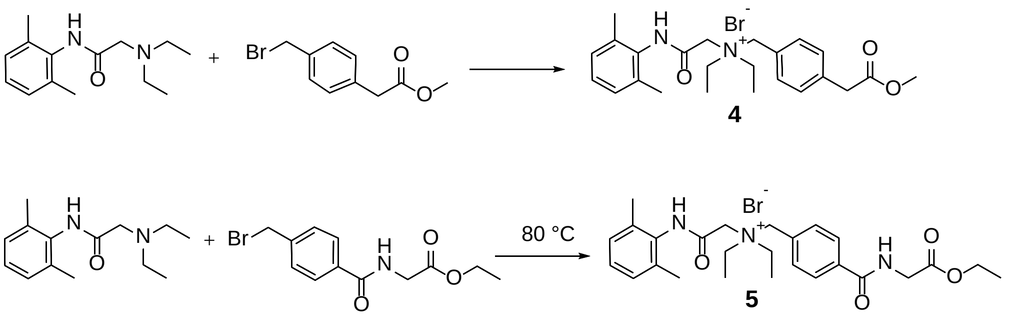

Supplementary Figure 4: Reaction of lidocaine with different bromomethylbenzoic acid esters and amides, respectively, in solid state.

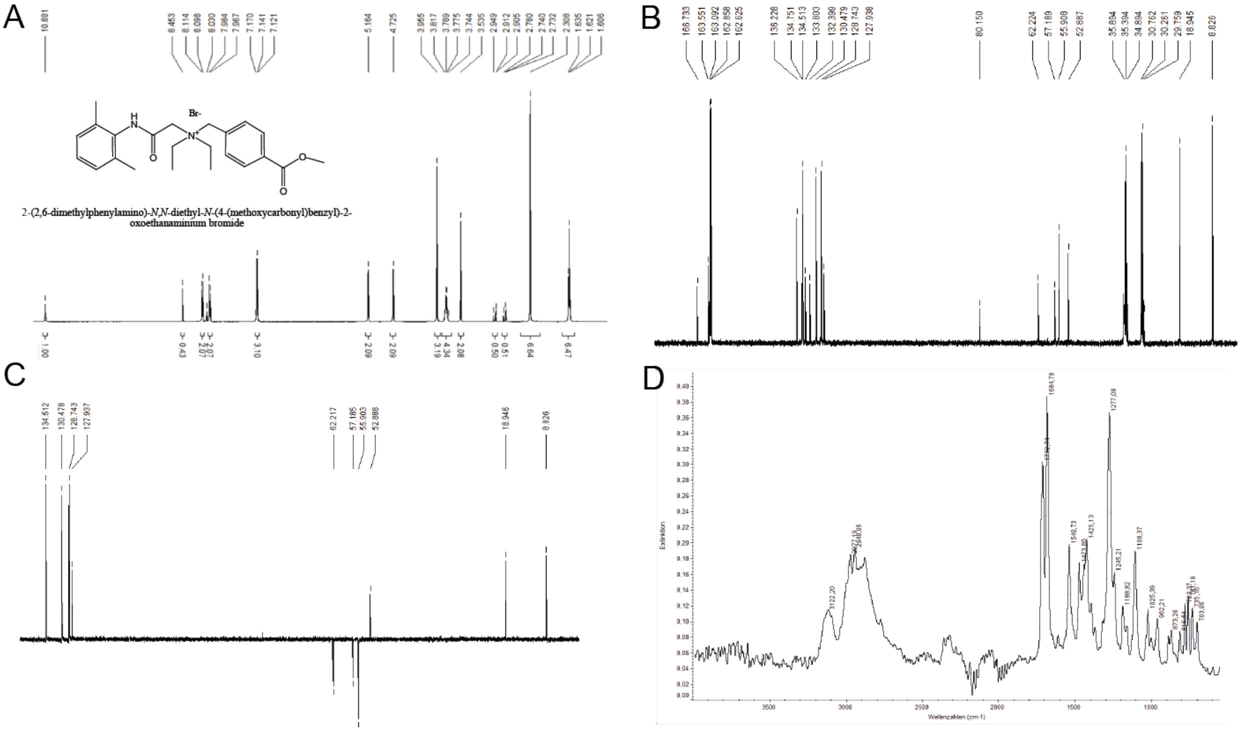

Supplementary Figure 5: Synthesis of 2-(2,6-dimethyl-phenylamino)-N,N-diethyl-N-(4-(methoxycarbonyl)benzyl)-2-oxoethanaminium bromide (1) A: 1H-NMR, B: 13C-NMR, C: 13C-DEPT-NMR, D: IR

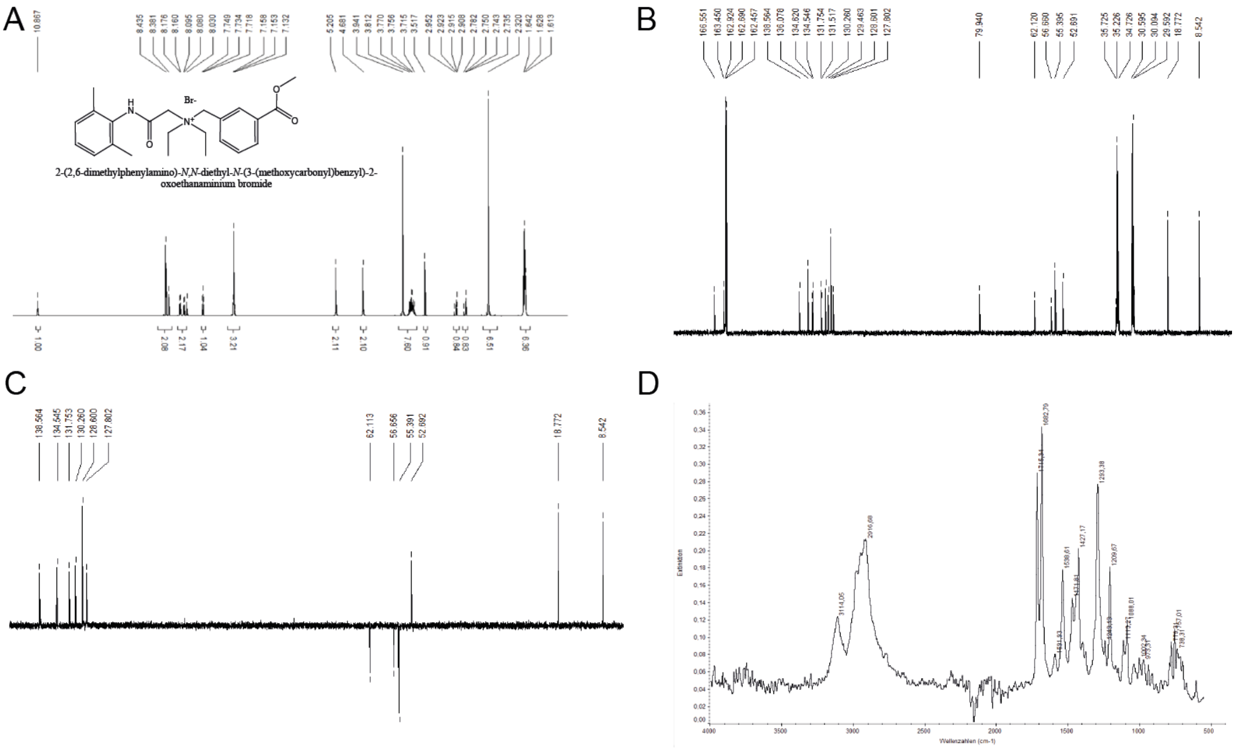

Supplementary Figure 6: Synthesis of 2-(2,6-dimethyl-phenylamino)-N,N-diethyl-N-(3-(methoxycarbonyl)benzyl)-2-oxoethanaminium bromide (2) A:1H-NMR, B: 13C-NMR, C: 13C-DEPT-NMR, D: IR

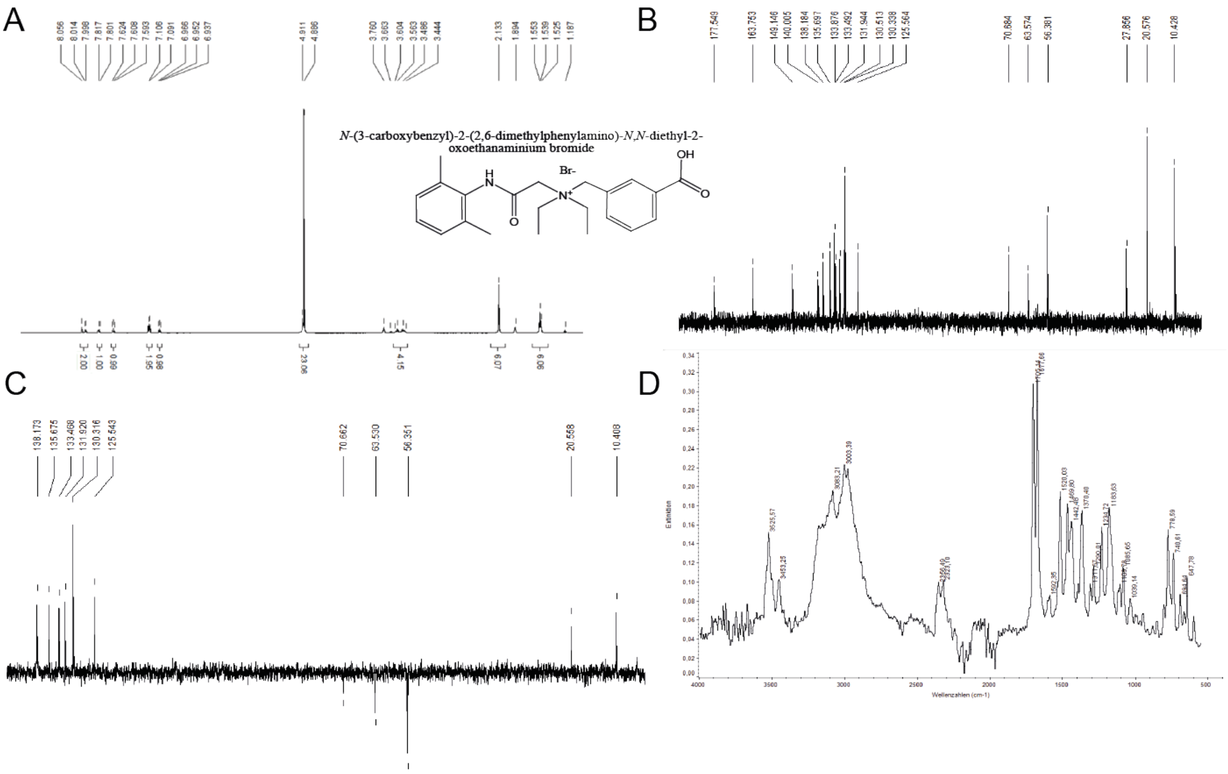

Supplementary Figure 7: Synthesis of N-(3-carboxybenzyl)-2-(2,6-dimethylphenylamino)-N,N-diethyl-2-oxoethanaminium bromide (3) A: 1H-NMR, B: 13C-NMR, C: 13C-DEPT-NMR, D: IR

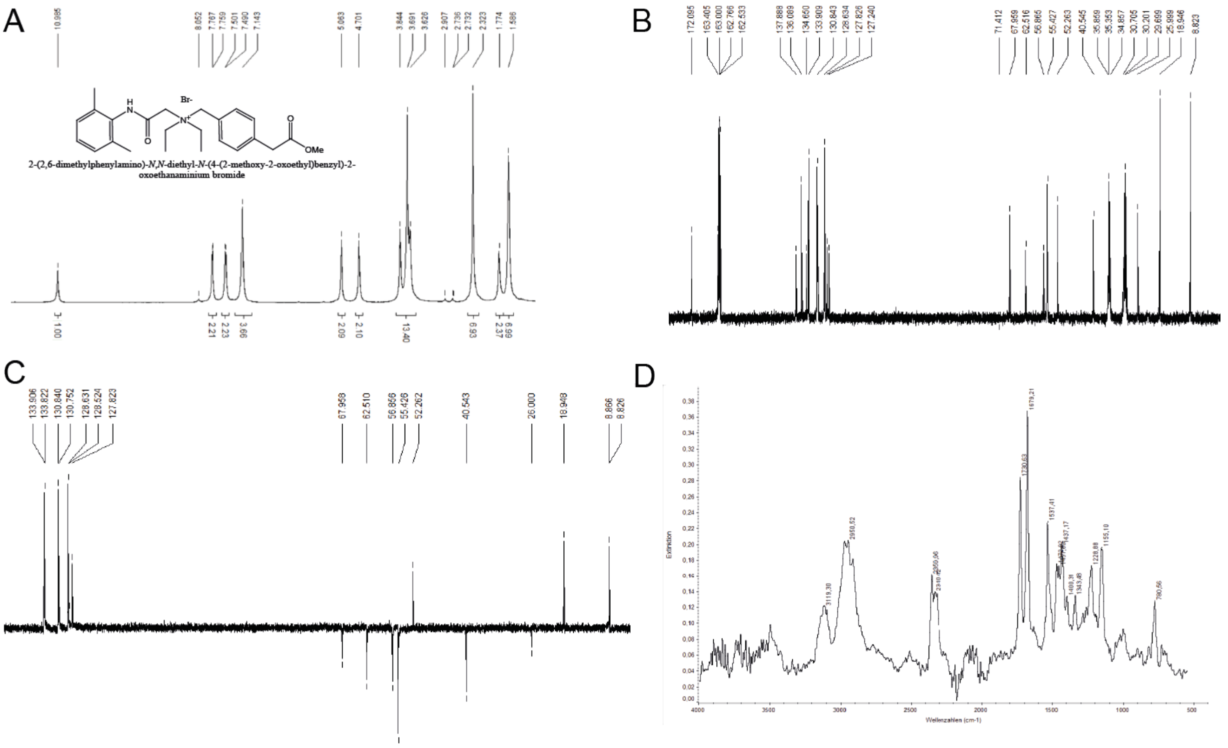

Supplementary Figure 8: Synthesis of 2-(2,6-dimethylphenylamino)-N,N-diethyl-N-(4-(2-methoxy-2-oxoethyl)benzyl)-2-oxoethanaminium bromide (4) A: 1H-NMR, B: 13C-NMR, C: 13C-DEPT-NMR, D: IR

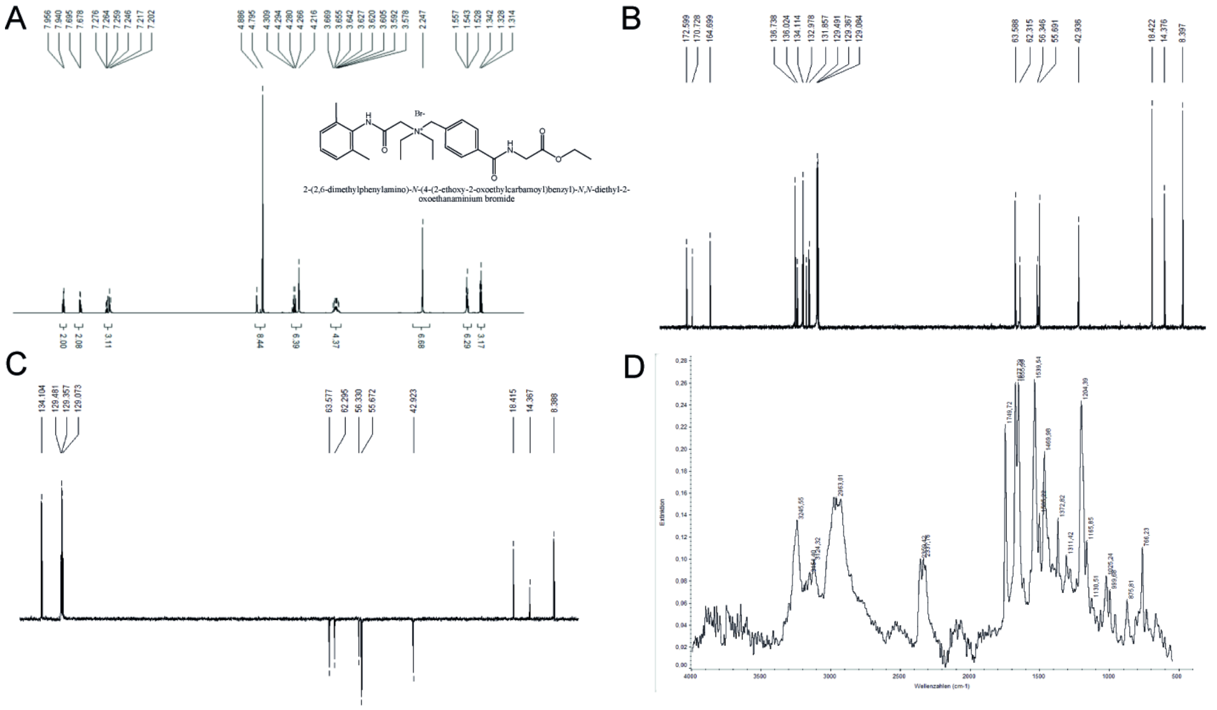

Supplementary Figure 9: Synthesis of 2-(2,6-dimethylphenylamino)-N-(4-(2-ethoxy-2-oxoethylcarbamoyl)benzyl) -N,N-diethyl-2-oxoethanaminium bromide (5)1H-NMR, B: 13C-NMR, C: 13C-DEPT-NMR, D: IR

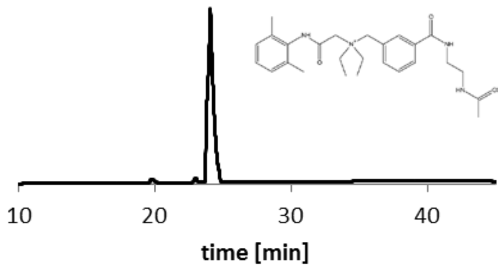

Supplementary Figure 10: (A) HPLC analysis of synthesized denatonium derivative carrying an acetylated diaminoethyl group (Diaminoethyl Denatonium acetylated; Diaminoethyl-D., Chemical structure is shown for clarification).

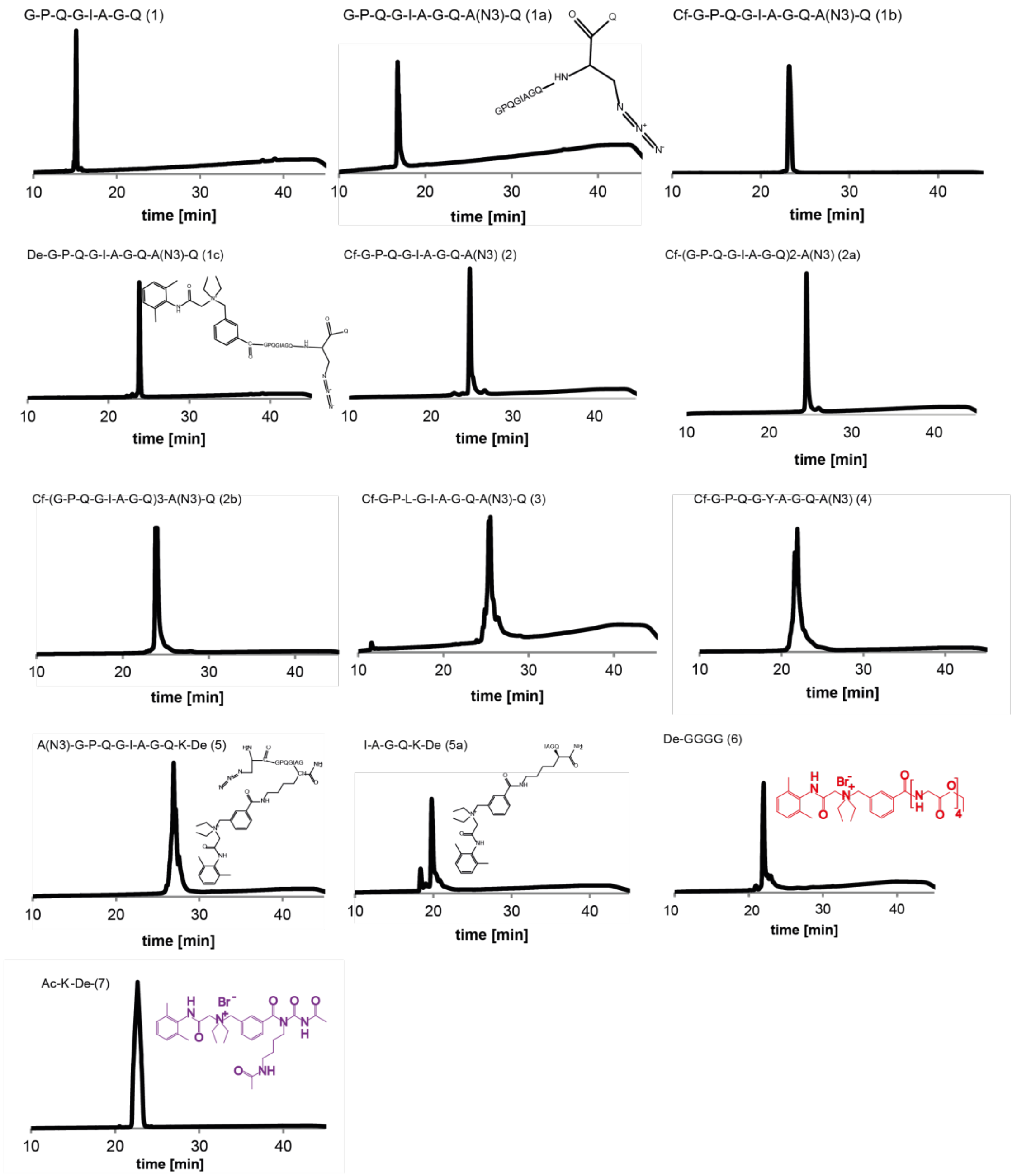

Supplementary Figure 11: HPLC analysis of PCL sequences and other peptides as outlined (**Table 1**). Peptides were characterized with HPLC as described. Complex chemical structures were shown for clarification.

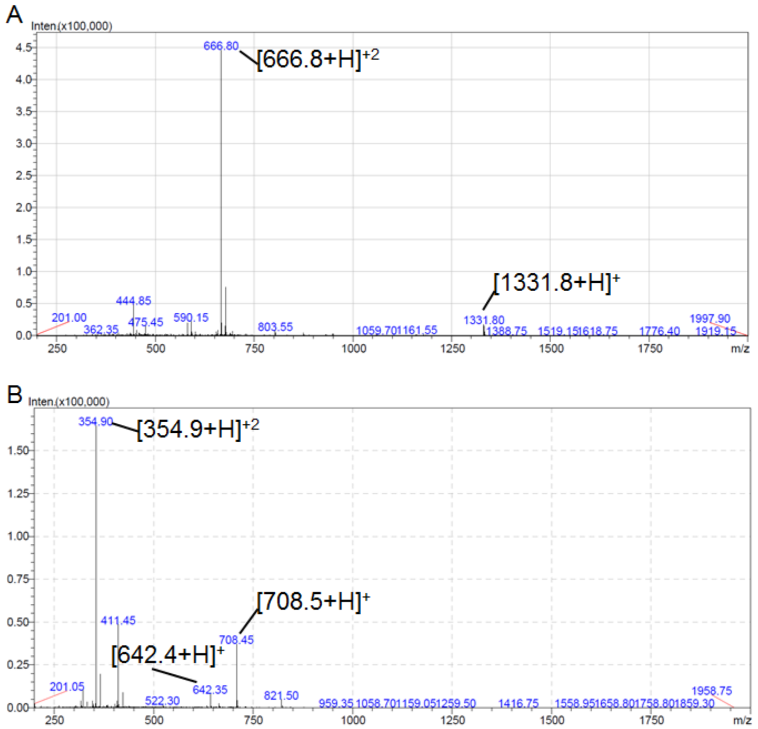

Supplementary Figure 12: (A) D.-CLAN$_3$Q (ID #1c) LC-MS analysis of negative control with a calculated mass of 1331 Da. (B) The MMP-8 cleavage products D.-GPQG and IAGQ(AN$_3$)Q eluting with the expected sizes that can be estimated by calculation (Mw = 642 and 708 Da).

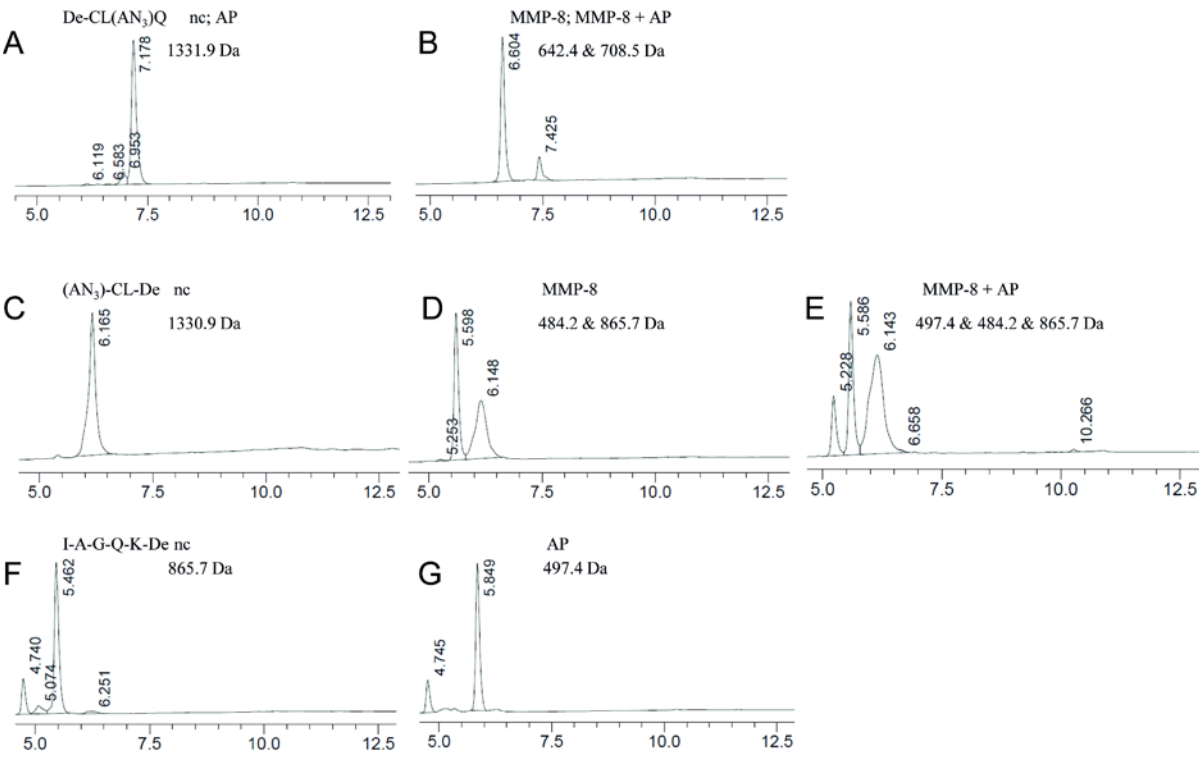

Supplementary Figure 13: (A) D.-CLAN$_3$Q (ID #1c) LC-MS analysis of negative control and AP-incubated peptide revealed either one HPLC-peak at 7.178 min with a mass of 1331.9 Da. (B) The MMP-8 cleavage products De-GPQG and IAGQ(AN$_3$)Q are clearly seen as two new peaks eluting with the expected sizes that can be estimated by calculation (Mw = 642 and 708 Da). An additional AP incubation doesn't change the outcome. (C) AN$_3$CL-D. (ID #5) negative control analysis by LC-

MS confirmed the calculated mass of 1331.1. (D) The MMP-8 cleavage products (AN_3)GPQG and IAGQK-De are clearly seen as two new peaks eluting with the expected sizes that can be estimated by calculation (Mw = 484 and 866 Da). (E) An additional peak appears after additonal AP-incubation (Mw = 497 Da (denatonium coupled lysin). (F) AP hydrolysis of fragment IAGQK-De (ID #5a) (Mw = 865.65 Da) results in (G) denatonium coupled to lysine (Mw = 497.4 Da). X-axis show time [min] and Y-axis intensity [mV]; Found molecular mass is provided in each spectra.

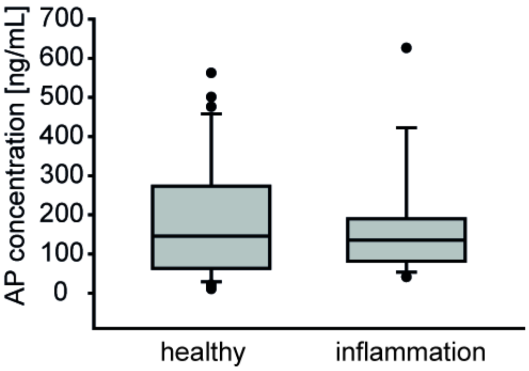

Supplementary Figure 14: Salivary aminopeptidase (AP) concentrations were determined in saliva collected from healthy donors (n = 36) and patients with inflammation in the oral cavity (n = 15). AP concentrations were 183 ± 155 ng/mL and 169 ± 143 ng/mL in healthy donors and patients, respectively. Outliers are shown as black dots. Box-and-whisker plots show the median (solid line), the box contains the middle 50 percent of values and the whiskers show the lower and upper 25 percent of the data, respectively.

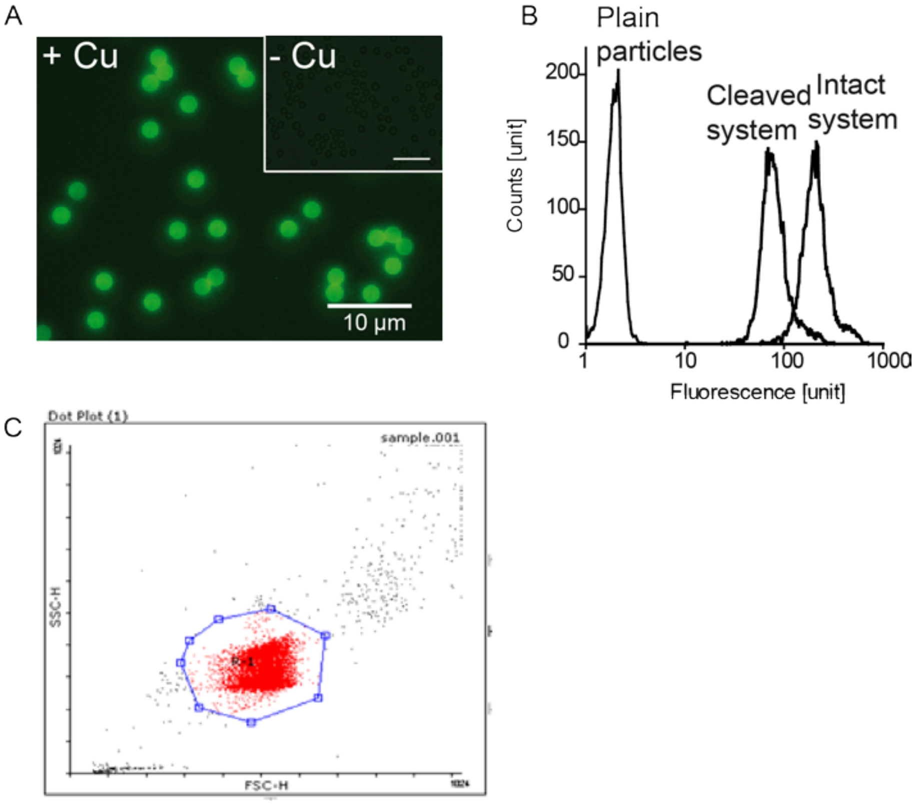

Supplementary Figure 15: (A) PMMA particles reacted with carboxyfluorescein decorated MMP-8 PCL (to form the sensor) in presence of Cu^{+} and without copper as indicated by fluorescence microscopy. (B) Quantitative FACS data of control particles, and particles reacted with carboxyfluorescein decorated MMP-8 PCL (sensor) before and after exposure to 900 ng/mL. (C) Analysis of fluorescence labeled PCL coupled to PMMA-beads (sensor). SSC vs. FSC density plot, carboxyfluorescein (λ_{Ex} = 492, λ_{EM} = 517 nm), FSC, forward scatter; SSC, side scatter.

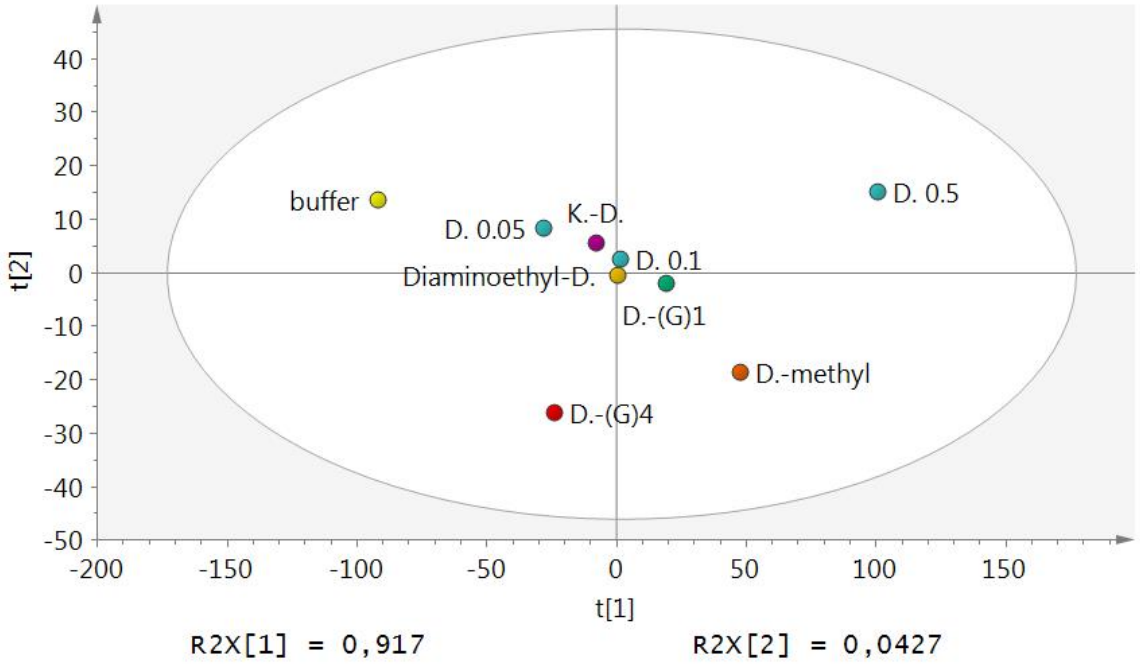

Supplementary Figure 16: Scores Scatter Plot containing the information of all applied bitter sensors; Denatonium benzoate (D.; blue, 0.05, 0.1 and 0.5 mM), Denatonium methyl ester (D.-methyl; orange, 0.5 mM), Denatonium (G)1 ester (D.-(G)1; green, 0.5 mM), Denatonium (G)4 ester (D.-(G)4; red, 0,5 mM), K-Denatonium (K.-D.; mauve, 0.5 mM), Diaminoethyl Denatonium acetylated (Diaminoethyl-D.; dark yellow, 0.5 mM), buffer (yellow). Data points are displayed as mean out of three subsequent measurements.

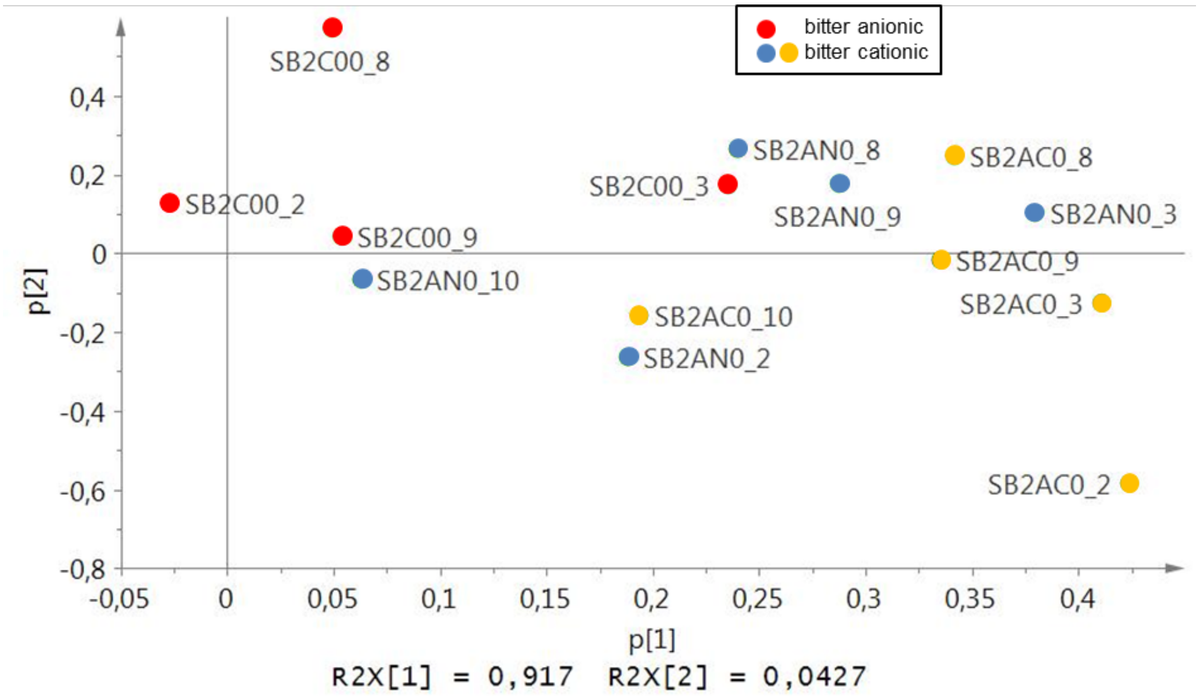

Supplementary Figure 17: Loading Scatter Plot; numbers within the labels are assigned to the different runs, in which the samples have been determined in. All experiments were performed with n = 3.

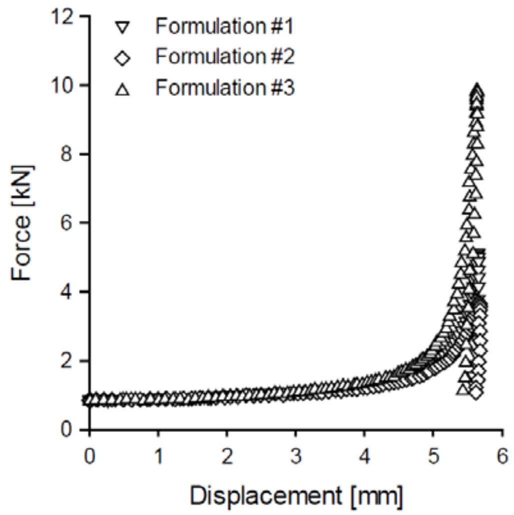

Supplementary Figure 18: Comparison of force-displacement diagrams of the HIG recipes.

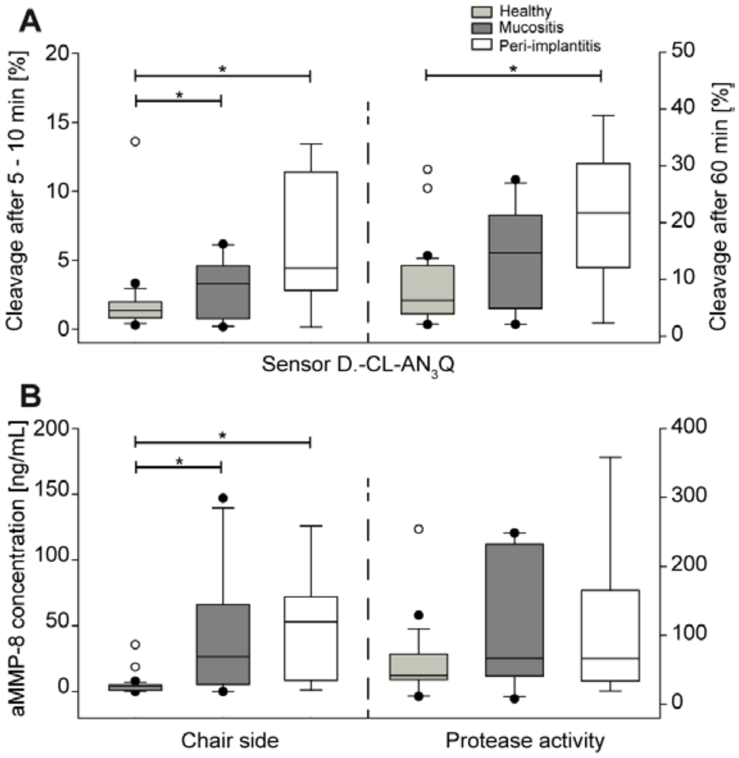

Supplementary Figure 19: Sensor performance in comparison to commercially available MMP diagnostics in patients with mucositis (n = 10), peri-implantitis (n = 9) and healthy control (n = 14). (A) Cleavage of the sensor in % after 5-10 and 60 minutes of incubation in saliva. (B) Active MMP-8 (aMMP-8) in sulcus fluid (chair-side test) and as measured from saliva (QuickZyme test). Outliers (not used for the analysis) are shown as white dots. Box-and-whisker plots show the median (solid line), the box contains the middle 50 percent of values and the whiskers show the lower and upper 25 percent of the data, respectively. Asterisks indicate a statistical difference ($p < 0.05$).

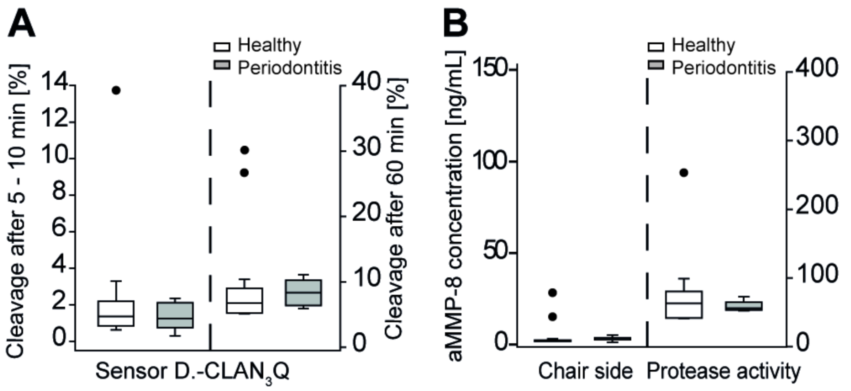

Supplementary Figure 20: Sensor performance in comparison to commercially available MMP diagnostics in periodontitis patients (n = 7) and healthy volunteers (n = 7). (A) Cleavage of the sensor in % after 5-10 and 60 minutes of incubation in saliva. (B) Active MMP-8 (aMMP-8) in sulcus fluid (chair-side test) and as measured from saliva (QuickZyme MMP-8 activity test). Outliers are shown as black dots and were not used for the analysis. No significant differences were observed among groups. Box-and-whisker plots show the median (solid line), the box contains the middle 50 percent of values and the whiskers show the lower and upper 25 percent of the data, respectively.

Supplementary Table 1: PCL sequences and other peptides characterization by MALDI-MS, and HPLC (Mass calc = calculated mass; Mass $[M+H]^{+1}$ = found mass, tR = retention time) *Multiple batch-batch variation

Peptide	Acronym	ID #	**Mass** calc.	**Mass** $[M+H]^{+}$	**tR** [min]	**Purity** [%]
**G-PQGIAGQ**-A-Q	CL-AQ	1	926.5	927.0	15.11	91.6
**G-PQGIAGQ**-A(N_3)-Q*	CL-AN$_3$Q	1a	981.5	982.3	16.95	78.2
Cf-**GPQGIAGQ**-A(N_3)-Q*	Cf-CLAN$_3$Q	1b	1324.2	1325.4	23.16	98.2
De-**GPQGIAGQ**-A(N_3)-Q*	D.-CLAN$_3$Q	1c	1331	1332.7	23.36	94.8
Cf-**GPQGIAGQ**-A(N_3)*	Cf-(CL)$_1$AN$_3$	2	1210.2	1212.1	24.13	98.2
Cf-(**GPQGIAGQ**)$_2$-A(N_3)	Cf-(CL)$_2$AN$_3$	2a	1919.1	1919.1	23.38	96.5
Cf-(**GPQGIAGQ**)$_3$-A(N_3)	Cf-(CL)$_3$AN$_3$	2b	2667.6	2667.2	23.54	99.1
Cf-GP**L**GIAGQ-A(N3)-Q		3	1309.2	1310.5	26.10	78.2
Cf-GPQG**Y**AGQ-A(N_3)-Q		4	1374.2	1375.6	22.24	56.4
A(N_3)-**GPQGIAGQ**-K-De*	AN$_3$-CL-D.	5	1331.1	1331	22.09	89.2
IAGQ-K-De *		5a	866.5	865.5	21.1	81.2
De-G-G-G-G	D.-(G)4	6	611.3	611.3	21.98	84.1
Ac-K-De *	K-D.	7	579.3	580.3	21.79	93.4

Supplementary Table 2: Summary of concentration dependent sensor signals; ++ = very sensitive (sensor signal increase or decrease of ≥ 41 mV), + = sensitive (sensor signal increase or decrease of 10-40 mV), (+) slightly sensitive (sensor signal increase or decrease of 4-10 mV), - = no dependency (sensor signal increase or decrease of ≤ 3 mV); investigated concentrations: 0.05-0.5 mM

		**SB2AC0** bitter cationic	**SB2AN0** bitter cationic	**SB2C00** bitter anionic	SB2AE1 astringent	SB2AAE umami	SB2CA0 sourness	SB2CT0 saltiness
Denatonium	De	++	+	(+)	+	+	+	(+)
Denatonium methyl ester	D.-methyl	++	+	-	+	+	+	-
Denatonium $(G)_1$ ester	D.-(G)1	++	+	+	+	(+)	(+)	-
Denatonium $(G)_4$ ester	D.-(G)4	+	-	+	+	(+)	-	(+)
K-Denatonium acetylated	K.-D.	(+)	-	(+)	++	-	-	+
Diaminoethyl Denatonium acetylated	Diaminoethyl-D.	(+)	+	(+)	++	(+)	+	+

Supplementary Table 3: Composition of several HIG-PWD-bases and recipes

HIG PWD-01		HIG PWD-02		HIG PWD-03	
gum base	22-26 %	gum base	28-32 %	gum base	33-37 %
xylitol	8-12 %	xylitol	8-12 %	sorbitol	4-8 %
softener	< 1.5 %	softener	< 1.5 %		
E-551 (silicon dioxide)	< 2.0 %	E-551 (silicon dioxide)	< 2.0 %	E-551 (silicon dioxide)	< 2.0 %
sorbitol	ad 100 %	sorbitol	ad 100 %	isomalt	ad 100%
recipe 01; (leading to formulation #1; *vide infra*)		recipe 02; (leading to formulation #2; *vide infra*)		recipe 03; (leading to formulation #3; *vide infra*)	
HIG PWD-01	97.5 %	HIG PWD-02	97.5 %	HIG PWD-03	97.5 %
aerosil®	1 %	aerosil®	1 %	aerosil®	1 %
magnesium-stearat	1.5 %	magnesium-stearate	1.5 %	magnesium-stearate	1.5 %

Supplementary References

1. William YS. Denatured alcohol. US3080326, (1963).
2. Dale E. Kaukeinen DE, Buckle AP, Paper presented at the Proceedings of the Fifteenth Vertebrate Pest Conference, Lincoln, NE (1992)
3. Payne HAS. Bitrex - A bitter solution to safety. Chem. Ind. 22, 721–723 (1988).
4. Sibert JR, Frude N. Bittering agents in the prevention of accidental poisoning: children's reactions to denatonium benzoate (Bitrex). Arch. Emerg. Med. 8(1):1-7(1991).
5. Berning CK, Griffith JF, Wild JE. Research on the effectiveness of denatonium benzoate as a deterrent to liquid detergent ingestion by children. Fundam. Appl. Toxicol. 2(1):44-48 (1982).
6. Janmejay R, Jayshree H, Ravindra N. Preparation of a quaternary ammonium hydroxide and use thereof for the preparation of a quaternary ammonium salt. NL5005520051130 (2005).
7. Saroli A. Interaction of Denatonium Chloride with the Bitter Taste Receptor. Z. Lebensm. Unters. Forsch. 180:227-229 (1985).
8. Stricklin GP, Jeffrey JJ, Roswit WT, Eisen AZ. Human skin fibroblast procollagenase: mechanisms of activation by organomercurials and trypsin. Biochemistry 22(1):61-68 (1983).
9. Byzia A, Szeffler A, Kalinowski L, Drag M (2016) Activity profiling of aminopeptidases in cell lysates using a fluorogenic substrate library. Biochimie 122:31-37.
10. Woertz K, Tissen C, Kleinebudde P, Breitkreutz J. Taste sensing systems (electronic tongues) for pharmaceutical applications. Int. J. Pharm. 417(1-2):256-271 (2011).
11. Vlasov Y, Legin A, Rudnitskaya A. Electronic tongues and their analytical application. Anal Bioanal. Chem. 373(3):136-146 (2002).
12. Anand V, Kataria M, Kukkar V, Saharan V, Choudhury PK. The latest trends in the taste assessment of pharmaceuticals. Drug discov. today 12(5-6):257-265 (2007).
13. Pein M, Gondongwe XD, Habara M, Winzenburg G. Interlaboratory testing of Insent e-tongues. Int. J. Pharm. 469(2):228-237 (2014).
14. Woertz K, Tissen C, Kleinebudde P, & Breitkreutz J A comparative study on two electronic tongues for pharmaceutical formulation development. J. Pharm. Biomed. Anal. 55(2):272-281 (2011).
15. Woertz K, Tissen C, Kleinebudde P, Breitkreutz J. Performance qualification of an electronic tongue based on ICH guideline Q2. J. Pharm. Biomed. Anal. 51(3):497-506 (2010).
16. United States Pharmacopeia 30 Monograph ‘Powder flow’. USP30 NF 25, Rockville, MD (2007).
17. Ehlers V, Helm S, Kasaj A, Willershausen B. The effect of Parodontax(R) on the MMP-8 concentration in gingivitis patients. Schweizer Monatsschrift fur Zahnmedizin = Revue mensuelle suisse d'odonto-stomatologie = Rivista mensile svizzera di odontologia e stomatologia, **121**(11)**:** 1041-1051 (2011).
18. Mantyla P, et al. Gingival crevicular fluid collagenase-2 (MMP-8) test stick for chair-side monitoring of periodontitis. J. Periodontal Res. 38(4):436-439 (2003).
